# Disinfectants

**Published:** 1866-08

**Authors:** 


### DISINFECTANTS.

SOME one says that noxious *effluvia* are absorbed in an incredibly short space of time, if two or three onions are cut in thin slices, and put on a plate, to be renewed every six hours. This is just as true as that the smarting from the scratch of a pin becomes instantaneously unfelt, if the person is knocked down. The only safe, healthful, and effectual method of keeping a sick-room "sweet" is, to keep everything scrupulously dry and clean; instantly remove every article of clothing or bedding which has an atom of dampness or moisture upon it, do not allow even pure water to stand a moment in the apartment, let the fireplace be always kept open, with a frequent and free admission of the pure and the fresh air from out doors. This should be done every two or three hours during the twenty-four. It is the pure air that sick people want, not an atmosphere loaded with the fumes of onions, for in a pint of air they displace just as many particles of fresh air as would burnt sugar, cologne-water, or the sulphureted hydrogen of the privy; for, be it remembered, it is not the odor which does the mischief, so much as the deficiency of nutritious particles of the atmosphere which it takes the place of. We should rather think, that every additional odoriferous article introduced into a sick-room only added to the difficulty, even though it were the perfumes from "Araby the Blest." The greatest humanity we can show to the sick is, to secure to them the most important remedies ever known, to wit, quietness, cleanliness, and pure air: these alone would cure three-fourths of all our diseases, but we will not use them; yet they are everywhere attainable, and cost nothing but a little trouble. With the same physicians and the same medicines, the mortality of the British army in the Crimea was diminished one-half, through the influence of FLORENCE NIGHTINGALE, in the procurement of greater comfort and cleanliness among the sick.

